# Cannabinoid-dependent potentiation of inhibition at eye opening in mouse V1

**DOI:** 10.3389/fncel.2014.00046

**Published:** 2014-02-19

**Authors:** Yury Garkun, Arianna Maffei

**Affiliations:** Department of Neurobiology and Behavior, The State University of New York–Stony Brook UniversityStony Brook, NY, USA

**Keywords:** endocannabinoids, synaptic plasticity, GABA, LTP, visual cortex, development

## Abstract

Cannabinoid (CB) signaling is a well established regulator of synaptic transmission. Recent work demonstrated that CB release is necessary for the induction of inhibitory synaptic plasticity. In primary visual cortex (V1) CB receptors are present throughout life, though their level of expression is developmentally regulated. In the input layer of V1 (layer 4, L4) these receptors show low levels of expression and colocalize with GABAergic terminals suggesting that they may play an important role in regulating GABAergic transmission. Here we show that in the developmental window extending from eye opening to the onset of the critical period for visual cortical plasticity L4 inhibitory inputs onto pyramidal neurons are highly sensitive to activation of CB release. More specifically, application of synthetic and endogenous CB receptors agonists led to a significant increase in the amplitude and frequency of both spontaneous inhibitory post-synaptic currents and miniature inhibitory post-synaptic currents. This form of inhibitory potentiation is activity-dependent, induced by repetitive bursting of pyramidal neurons and regulated by the time of eye opening. CB-dependent regulation of inhibitory drive may be a mechanism for the regulating L4 pyramidal neurons excitability and function at a time in which V1 transitions from being activated by spontaneous activity to being driven by visual inputs.

## INTRODUCTION

The cannabinoid (CB) signaling pathway is determinant for the development of neural circuits from very early in life (Fride, 2004). In addition, CB signaling has been implicated in a variety of forms of synaptic plasticity that contribute to experience-dependent postnatal refinement of neocortical circuits (Chevaleyre and Castillo, 2004). CB receptor expression is tightly developmentally regulated, and in the primary visual cortex (V1), the best studied model for experience-dependent plasticity, it changes over the course of the first few postnatal weeks (Yoneda et al., 2013). In the input layer of V1 CB receptors are expressed at low levels throughout development and colocalize with GABAergic terminals (Yoneda et al., 2013). Previous work showed that inhibitory synapses are highly plastic in response to changes in visual drive both before and after the opening of the critical period for visual cortical plasticity (Maffei et al., 2004; Maffei and Turrigiano, 2008; Maffei et al., 2010). The presence of CB receptors specifically on GABAergic terminals during postnatal development raises the possibility that they may contribute significantly to the experience-dependent refinement of the circuit in the input layer of V1. To date CB signaling has been shown to be involved in long term depression of excitatory and inhibitory synapses (Hashimotodani et al., 2007; Castillo et al., 2012), but our understanding of CB role in neural circuit postnatal development remains rather limited.

Here we show that CB signaling is required for the induction of a novel form of inhibitory potentiation that is induced specifically during the developmental window between eye opening and the onset of the critical period. CB receptor agonists increased the amplitude and frequency of both spontaneous inhibitory post-synaptic currents (sIPSCs) and miniature inhibitory post-synaptic currents (mIPSCs) onto layer 4 pyramidal neurons. This effect was mimicked by repetitive burst firing of pyramidal neurons, suggesting that this form of CB-dependent inhibitory plasticity is activity-dependent, and may contribute to regulating pyramidal neuron excitability. Because of the specificity of this form of plasticity for the pre-critical period we asked whether it could be modulated by the time of eye opening. Delaying eye opening with binocular eyelid suture did not impair the maturation of inhibitory inputs, but reopened the window of sensitivity to CB signaling, suggesting that this form of inhibitory plasticity may play a crucial role in regulating circuit excitability at a developmental time in which V1 transitions from being activated by spontaneous activity to being driven by visual stimuli. Eye opening is characterized by a transient increase in spontaneous retinal activity (Stasheff et al., 2011) that may lead to strong activation of cortical neurons. A global increase in inhibitory synaptic drive induced by repetitive pyramidal neuron bursting like the one we report here could allow the network to control its excitability in the face of increased sensory drive. Thus, the data we report propose a new role for CB signaling in cortical circuits: that of regulator of cortical circuit excitability during early postnatal development.

## MATERIALS AND METHODS

The experiments were performed on C57BL/6 mice with age ranging from postnatal day 13 (P13) to P27. All experimental procedures were approved by the Stony Brook University Animal Use Committee and followed the guidelines of the National Institute of Health.

### DELAYED EYE OPENING

In a group of animals (*n* = 4) binocular lid suture was performed on the day before eye opening (P13). Animals were anesthetized with a cocktail of 70 mg/kg ketamine, 3.5 mg/kg xylazine hydrochloride, and 0.7 mg/kg acepromazine maleate, injected intraperitoneally. The area surrounding the eyes was cleaned with betadine, eye drops were administered to maintain a good level of eye moisture, and the eyes were covered with a thin layer of xylocaine gel (Maffei et al., 2004, 2010). Lids were closed with three or four mattress sutures using 6-0 polyester (Ethicon, Somerville, NJ, USA). Eyelid sutures were checked every day to ensure complete binocular deprivation until P27.

### SLICE PREPARATION

Coronal slices containing primary visual cortex (V1) were obtained from both hemispheres. Slices were incubated at room temperature in artificial cerebrospinal fluid (ACSF) containing (in mM): NaCl 126, KCl 3, NaH_2_PO_4_ 1, NaHCO3 25, MgSO_4_ 2, CaCl_2_ 2, dextrose 14 (318–320 mOsm). Recordings were performed at 32 ± 0.5°C in ACSF. Eyelid suture and slice preparation were blind to the experimenter.

### ELECTROPHYSIOLOGY

Patch clamp recordings were performed from visually identified neurons within layer 4 (Maffei et al., 2004, 2006, 2010). The resistance of recording electrodes was 3–4 MΩ when filled with the internal solution containing (in mM): KCl 120, HEPES 10, EGTA 0.5, Mg-ATP 4, Na-GTP 0.3, phosphocreatine 10. The osmolarity of the internal solution was adjusted to 290 mOsm with sucrose and pH was adjusted to 7.2–7.3 with KOH. Signals were acquired using HEKA EPC 10 amplifier and PatchMaster software (both from HEKA Elektronik, Germany). Neurons were identified based on their response to depolarizing current pulses of increasing amplitude (25 pA increments). Neurons that were included in the analysis met the following criteria: membrane potential (V_m_) lower than -65 mV, input resistance (R_in_) above 120 MΩ and series resistance below 15 MΩ. Neurons were included in the analysis if these parameters did not change more than 10% during recordings. sIPSCs were pharmacologically isolated by adding the α -amino-3-hydroxy-5-methyl-4-isoxazolepropionic acid (AMPA) receptor blocker 6,7-dinitroquinoxaline-2,3-dione (DNQX; 20 μM) and the *N*-methyl-D-aspartate (NMDA) receptor antagonist D-(-)-2-amino-5-phosphonopentanoic acid (APV; 50 μM) to the bath solution. mIPSCs were recorded in the presence of DNQX (20 μM), APV (50 μM) and TTX (0.1 μM). Spontaneous excitatory post-synaptic currents (sEPSCs) were isolated by adding picrotoxin (20 μM) to the regular ACSF solution. Neurons were recorded in voltage clamp at -70 mV. After acquisition of a stable baseline (10 min), ACSF containing a drug of interest was perfused in the recording chamber. Synaptic events were then monitored for at least 20 min following drug perfusion. The 150 events right before initiating drug perfusion were compared to the 150 events recorded 10 min following drug application. All data analysis was performed off-line using custom macros written in IGOR Pro (Wavemetrics, Lake Oswego, OR, USA). Distributions of sIPSC and sEPSC amplitudes/frequencies included 150 events/neuron.

### INDUCTION PARADIGM

Long depolarizing current steps have been used to reliably modulate the strength of inhibitory inputs (Pitler and Alger, 1992; Lenz et al., 1998; Kreitzer and Regehr, 2001; Ohno-Shosaku et al., 2001; Wilson and Nicoll, 2001; Hampson et al., 2003; Mato et al., 2004; Lemtiri-Chlieh and Levine, 2007). While depolarizing stimuli are mostly inducing a transient depression of IPSCs we tested the possibility that in L4 of V1 in the pre-critical period a similar paradigm could contribute to the CB-dependent potentiation we report. To do that we recorded pyramidal neurons in voltage clamp in the presence of AMPA and NMDA receptor blockers to isolate inhibitory currents. After a few minutes baseline acquisition we injected either a 3s or a 5s square depolarizing pulse in the recorded neuron to drive its membrane potential from -70 to 0 mV (Ohno-Shosaku et al., 2001; Chevaleyre and Castillo, 2004). As this paradigm was ineffective we explored the possibility that post-synaptic spiking may be necessary for mimicking the potentiation of sIPSCs induced by WIN and anandamide.

To test whether pyramidal neuron spiking can mimic the effect of CB agonists on sIPSCs amplitude and frequency we tested three different paradigms. All these sets of inductions were performed in the presence of AMPA and NMDA receptor blockers; therefore no recurrent excitatory circuit activity was engaged by the induction paradigms. (1) Pyramidal neurons were depolarized above threshold with a single 3 ms long depolarizing current step in current clamp. (2) A single set of 20, 3 ms long suprathreshold depolarizing current steps tightly timed at 50 Hz was used to elicit one burst of action potentials (APs) in the recorded pyramidal neuron (Fortin et al., 2004; Lemtiri-Chlieh and Levine, 2007). (3) A series of 20 bursts composed of 10, 3 ms long suprathreshold depolarizing current steps at 50 Hz, was delivered at 0.1 Hz to mimic repetitive pyramidal neurons bursting in the slow frequency range (Wang et al., 2012).

### STATISTICAL ANALYSES

All data are presented as mean ± SEM. Statistical significance was determined with two-tailed paired *t*-tests to assess the effect of a drug within a recorded neuron and unpaired *t*-tests to compare across neurons. Bonferroni’s correction was applied for multiple comparisons. Differences in amplitude and frequency of sIPSC across development were tested by one-way ANOVA and followed by a Tukey–Kramer multiple comparison tests. Kolmogorov–Smirnov (K–S) tests were used to assess differences in the cumulative distribution of amplitude and frequency of synaptic events. *P* values ≤ 0.05 were considered significant.

### DRUGS

All drugs were diluted in ACSF to the appropriate concentration. In the experiments presented here the drug used were: the sodium channel blocker tetrodotoxin (TTX; 0.1 μM); the NMDA receptor antagonist D-(-)-2-amino-5-phosphonopentanoic acid (APV, 50 μM; Tocris); the AMPA/kainate receptor antagonist (DNQX, 20 μM; Sigma); the synthetic CB receptor agonist (R)-(+)-[2,3-dihydro-5-methyl-3-(4-morpholinylmethyl)pyrrolo[1,2,3-de]-1,4-benzoxazin-6-yl]-1-naphthalenylmethanone mesylate (WIN 55,212-2; 1 μM); the endogenous CB agonist *N*-(2-hydroxyethyl)-5Z,8Z,11Z,14Z-eicosatetraenamide (Anandamide, 1 μM); the CB_1_ receptor antagonist 1-(2,4-dichlorophenyl)-5-(4-iodophenyl)-4-methyl-N-4-morpholinyl-1H-pyrazole-3-carbox-amide (AM281, 1 μM); the selective vanilloid receptor antagonist *N*-[2-(4-chlorophenyl)ethyl]-1,3,4,5-tetrahydro-7,8-dihydroxy-2H-2-benzazepine-2-carbothioamide (Capsazepine, 5 μM); the opioid receptor agonist DAMGO (0.1 μM); the neurotrophin brain-derived neurotrophic factor (BDNF; 20 ng/ml); the calcium chelator 1,2-bis(2-aminophenoxy)ethane-N,N,N’,N’-tetraacetic acid (BAPTA). All chemicals were obtained from Tocris.

## RESULTS

During early postnatal development CB receptors colocalize with GABA releasing axon terminals in the input layer of V1, suggesting a role for CB signaling in the postnatal maturation of inhibitory inputs (Yoneda et al., 2013). To identify the effect of CB on inhibitory synapses whole-cell patch clamp recordings were performed from layer 4 pyramidal neurons in V1. sIPSCs were pharmacologically isolated by bath application of the ionotropic glutamate receptor antagonists DNQX (20 μM) and APV (50 μM). In a subset of experiments the sodium channel blocker tetrodotoxin (TTX, 0.1 μM) was also added to the bath and the amplitude and frequency of mIPSC was compared to that of sIPSCs recorded in the absence of TTX. No significant differences were observed between these conditions (not shown) indicating that in our preparation sIPSCs do not result from spontaneous spiking activity in the slice and can be well approximated to mIPSC. Most of the results presented in the manuscript were obtained recording sIPSCs. In a subset of experiments mIPSC were recorded to assess whether WIN could directly affect them as well. A KCl-based internal solution was used to set the reversal potential of chloride to 0 mV in order to improve the detection of mIPSCs and sIPSCs at the holding potential of -70 mV. In these experimental conditions inhibitory events were recorded as inward currents. Amplitude, frequency and kinetic of mIPSCs, sIPSCs and sEPSCs were measured to quantify the effect of CB on inhibitory synaptic currents.

### THE CB AGONIST WIN 55,212-2 POTENTIATES INHIBITION IN LAYER 4 OF V1

**FIGURE 1:**
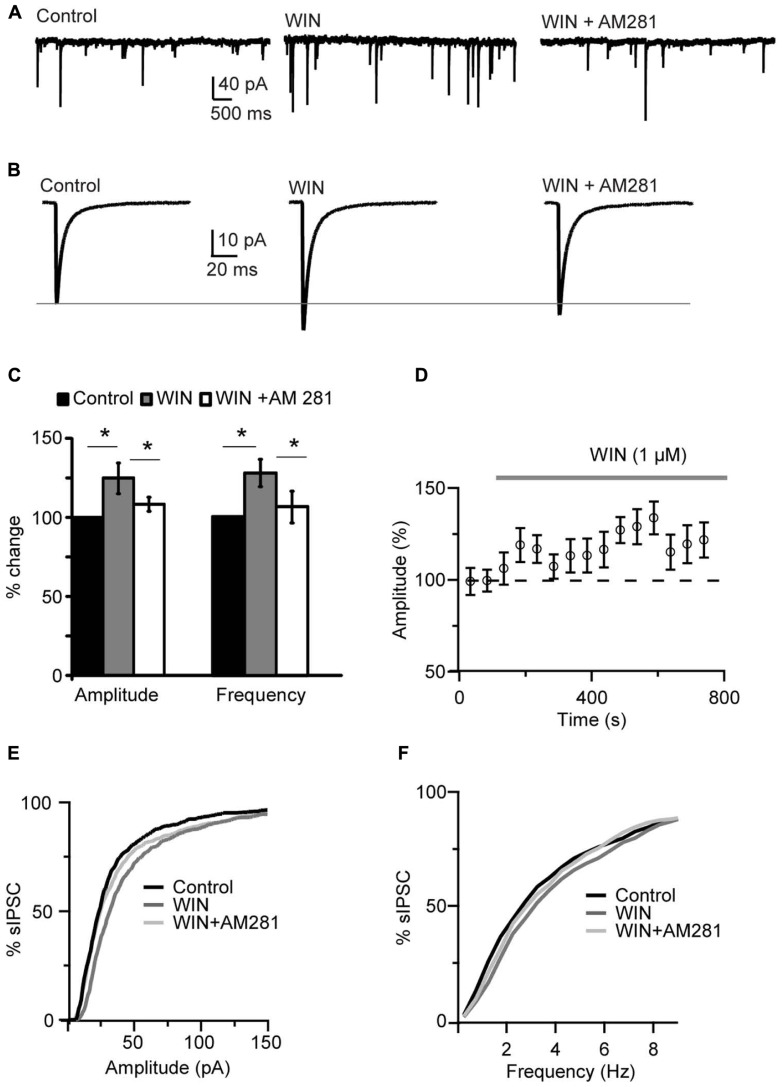
** WIN 55,212-2 potentiated inhibitory synaptic transmission in layer 4 of V1. (A)** Representative recordings of sIPSCs before (Control), during perfusion of WIN 55,212-2 (WIN, 1 μM) and during perfusion of AM281 (WIN+AM281, 1 μM each). All traces were obtained from the same cell at holding potential -70 mV. **(B)** sIPSC traces averaged across 150 events for the same conditions shown in **(A)**. **(C)** Mean amplitude and frequency of sIPSCs increased after WIN 55,212-2 application. The effect was reversed by the CB1 antagonist AM281. Amplitude and frequency are represented relative to Control to show fold changes. Black: control; gray: WIN 55,212-2; white: WIN 55,212-2 + AM281). **(D)** Time course of sIPSC recordings. The gray bar indicates the presence of WIN 55,212-2 in the bath. **(E,F)** Cumulative distributions of sIPSC amplitude **(E)**, and frequency **(F)**. Black: control; dark gray: WIN 55,212-2; light gray: WIN 55212-2 + AM281. Data are presented as mean ± SEM, statistical significance is indicated by * for *P* < 0.05.

Bath application of the CB agonist WIN 55,212-2 (1 μM) significantly increased sIPSCs amplitude and shifted the cumulative amplitude distribution of sIPSCs toward higher values (125.0 ± 9.5% of baseline, paired *t*-test (p-*t*) on average data: *P *< 0.04; K–S test on distribution: *P* < 0.001;* n *= 15; **Figures 1A–E**). The mean sIPSC frequency was also increased (127.1 ± 8.4% of baseline; p-*t* on average data: *P *< 0.004; K–S test on distribution: 0.001; **Figure 1C**). The effect of the agonist persisted throughout the period of WIN 55,212-2 exposure (**Figures 1C,D,F**). WIN 55,212-2 application did not affect resting membrane potential (p-*t*:* P* = 0.6, *n*= 15), input resistance (*P* = 0.4) and series resistance (*P* = 0.3) of recorded neurons.

Following WIN 55,212-2 application the CB1 receptor antagonist AM281 (1 μM) was bath applied to determine the specificity of the effect of WIN on mIPSCs. AM281 reversed the effect of WIN 55,212-2, returning mean sIPSC amplitude and frequency to baseline values (amplitude, 104.4 ± 4.4%; frequency, 105.7 ± 10.0%; p-*t* WIN vs. AM281, amplitude: *P* < 0.05 and frequency: *P* < 0.05, *n*= 5; p-*t* AM281 vs. baseline, amplitude: *P* = 0.3 and frequency: *P* = 0.86; *n*= 5; **Figures 1A–C**). Application of AM281 also shifted the cumulative amplitude and frequency distributions of sIPSCs back toward control levels (K–S test on distributions, WIN vs. AM281, amplitude: *P* < 0.001; frequency: *P* < 0.001). These data strongly suggest that the modulation of sIPSC we observed was due to CB1 receptor activation. WIN 55,212-2 significantly increased also mIPSC amplitude and frequency in recordings in which 0.1 μM TTX was bath applied, indicating that the effect of WIN does not depend on recurrent circuit activation (amplitude: 109.2 ± 3.4% of control; p-*t*: *P* < 0.04; frequency 121.1 ± 4.8% of control; p-*t*: *P* < 0.02; *n*= 7). To further verify that the effect of WIN 55,212-2 was specific to GABA_A_ inputs, the GABA_A_ antagonist picrotoxin (30 μM) was bath applied at the end of each experiment. Picrotoxin abolished all synaptic events (sIPSC frequency in picrotoxin: 0.001 ± 0.02 Hz, *n*= 5, *P* < 0.0007, paired *t*-test).

**FIGURE 2:**
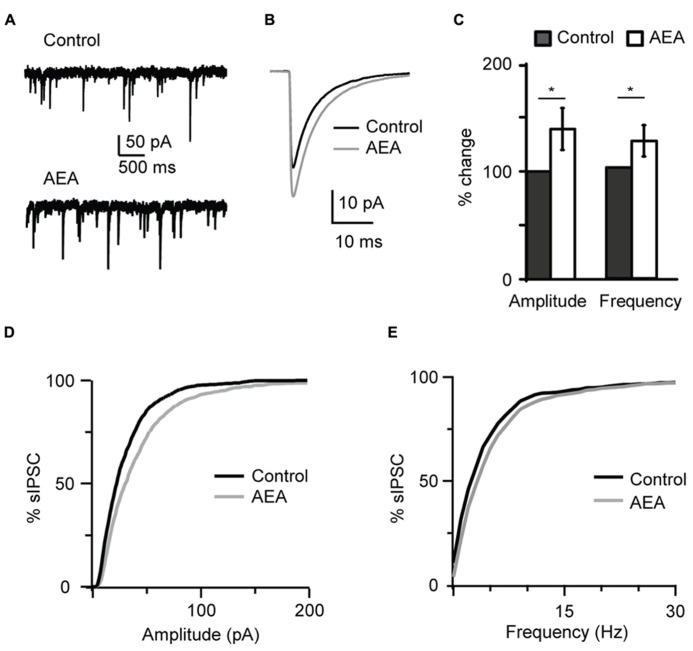
** The endogenous CB agonist AEA (anandamide) replicated the effect of WIN 55,212-2 on inhibitory neurotransmission. (A)** Example traces of sIPSCs in the absence (Control) and in presence of AEA (1 μM). **(B)** sIPSC traces averaged across 150 events before (black) and during the perfusion of AEA (gray) for the cell shown in **(A)**. **(C)** Bar plot summarizing the effect of AEA on sIPSC amplitude and frequency. Amplitude and frequency are represented relative to Control to show fold changes. Black: control; white: AEA. **(D,E)** Cumulative distributions of sIPSC amplitude **(D)**, and frequency **(E)** in control (black), and in the presence of AEA (gray). Data are presented as mean ± SEM, statistical significance is indicated by * for *P* < 0.05.

In order to determine whether CB-dependent potentiation of inhibition is mediated by agonists that can be released endogenously *in vivo*, we asked if this effect could be replicated by a non-synthetic CB receptor ligand. Application of 1 μM anandamide (AEA), an endogenous CB receptor agonist, recapitulated the effect of WIN 55,212-2 by increasing the amplitude and frequency of sIPSC recorded at P19 (amplitude, 138.9 ± 19.4%; frequency, 124.0 ± 14.1%, *n*= 7; K–S test on distributions: *P* < 0.001 for both parameters; p-*t* on average data:* P *< 0.05 for both parameters; **Figure 2**).

**FIGURE 3:**
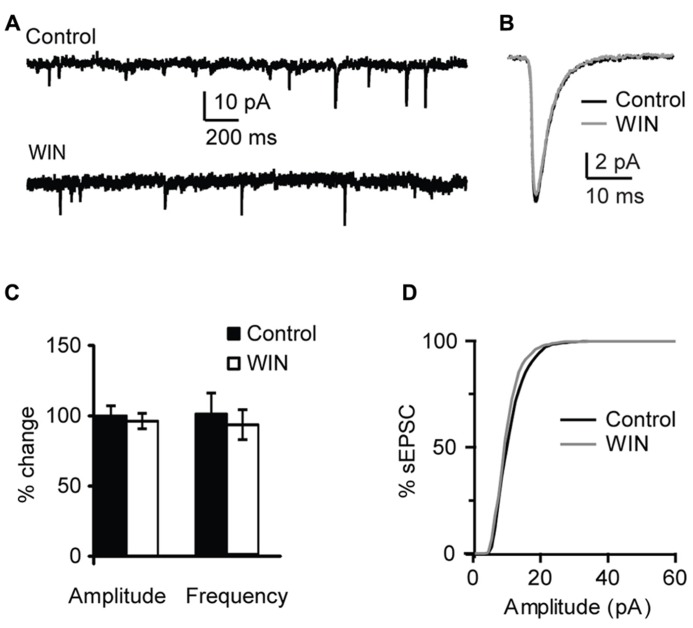
** WIN 55,212-2 does not affect mEPSCs in layer 4 of V1. (A)** Example traces of mEPSCs recordings before (Control) and during bath application of WIN 55,212-2 (WIN, 1 μM). **(B)** mEPSC traces averaged across 150 events for the same conditions shown in **(A)**. Black: control; gray: WIN. **(C)** Bar-plot summarizing the effect of WIN 55,212-2 on mEPSC amplitude and frequency. Black: control; white: WIN 55,212-2. Amplitude and frequency are represented relative to the Control to show fold changes. **(D)** Cumulative distribution of mEPSC amplitude in control (black) and in the presence of WIN 55,212-2 (gray).

This effect was specific to GABAergic inputs. In a subset of experiments sEPSCs were recorded and the effect of WIN 55,212-2 application on excitatory events was quantified. Recordings were performed with the internal solution where 120 mM KCl were replaced with a mixture of 20 mM KCl and 100 mM K-gluconate in order to improve the detection of sEPSC at the holding potential of -70 mV. Excitatory events were recorded in the presence of picrotoxin (20 μM). There was no change in sEPSC amplitude and frequency following application of 1 μM WIN 55,212-2 (amplitude: 96.3 ± 5.4 % of baseline, p-*t* on average data: *P* = 0.68; K–S test on distribution: *P* = 0.66; frequency: 92.4 ± 10.5 % of baseline, p-*t* on average data: *P* = 0.39; K–S test on distribution: *P* = 0.97; *n* = 10; **Figure 3**). These results indicate that in L4 of V1 CB signaling selectively enhanced GABA_A_ receptors-mediated inhibitory drive onto pyramidal neurons.

**FIGURE 4:**
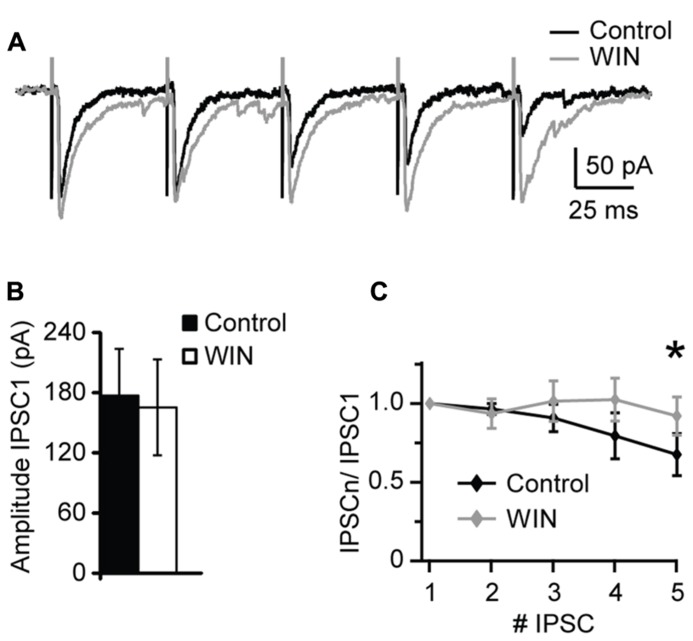
** WIN 55,212-2 modulates short-term dynamics of evoked IPSCs. (A)** Sample traces of evoked IPSP recorded before (Control; black) and in the presence of WIN 55,212-2 (WIN, 1 μM, gray). **(B)** Average data of IPSC1 amplitude before and after WIN 55,212-2 application. Black bar: control; white bar: WIN. **(C)** Average plot of short-term changes in IPSP amplitude in response to five stimuli at 20 Hz. Data are presented as ratio of the first IPSP in the train. Black: control; gray: WIN. Data are presented as mean ± SEM. Statistical significance is indicated by * for *P* < 0.05.

Changes in sIPSCs and mIPSCs frequency suggest a presynaptic site of expression of the CB-dependent potentiation of inhibition we report. To investigate that more thoroughly we used extracellular stimulation to evoke IPSCs with repetitive stimuli and quantify possible changes in short-term dynamics. A bipolar tungsten stimulating electrode was placed within L4 close to the recorded neuron (20–40 μm from recorded soma). Stimuli (5 × 0.2 ms pulses at 20 Hz) were delivered through a stimulus isolation unit driven by the acquisition board built in into our HEKA amplifier. At the beginning of each experiment an input/output curve was established for each neuron to determine the intensity of the stimulation that evoked IPSC reliably in the mid-range of the curve. This intensity was used throughout the recording. After acquisition of a 10 min baseline WIN 55,212-2 (1 μM) was bath applied and evoked IPSCs recorded for several minutes. The amplitude and short-term dynamics of evoked baseline IPSCs were compared with those recorded in the presence of WIN 55,212-2. As shown in **Figure 4**, there was no significant change in the amplitude of the first evoked IPSC in the train following application of WIN 55212-2. However, the short-term dynamics of IPSCs in response to repetitive stimulation were significantly affected (**Figure 4C**). While the paired pulse ratio was not significantly different (*P* = 0.4, p-*t*), in the presence of WIN 55212-2 evoked IPSCs showed less short-term depression, consistent with an increase in the reliability of inhibitory synaptic transmission in response to repetitive activation of inhibitory inputs (IPSP5/IPSP1 control, 0.67 ± 0.13; IPSP5/IPSP1 after WIN 55,212-2, 0.92 ± 0.12, *n* = 8, p-*t*:* P* < 0.05; **Figure 4C**). Taken together, these results show that CB release can potentiate inhibitory synaptic responses by activating CB signaling pathways and increasing the reliability of GABAergic release during repetitive stimulation. In the hippocampus it was reported that GABAergic synapses GABA_A_ receptors are not saturated by a single release event (Cohen et al., 2000). While this has not been directly studies in V1, a similar mechanism may explain our results in L4. A possible interpretation of our data is that an increase in the reliability of synaptic transmission by up-regulation of presynaptic release could explain the increase in both amplitude and frequency of sIPSCs and mIPSCs following WIN 55212-2 application.

### POTENTIATION OF INHIBITORY SYNAPTIC TRANSMISSION IS SPECIFIC TO CB-SIGNALING

The effect of CB signaling is mostly associated with long term depression both at excitatory and inhibitory synapses (Chevaleyre et al., 2006; Hashimotodani et al., 2007; Castillo et al., 2012). To date only two reports have shown evidence for CB-dependent potentiation of synaptic transmission, one that investigated electrical and chemical excitatory synapses onto Mauthner cells in the goldfish (Cachope et al., 2007), and one that tested the effect of CB agonists on AP-independent plasticity of inhibitory synapses in rat hippocampus (Hofmann et al., 2011).

Recent findings suggest that CB could activate non-CB targets, such as the transient receptor potential TRPV1, as well as opioid and BDNF receptors. Thus, we designed experiments to investigate whether the potentiation of inhibition we observe is specific for CB targets.

**FIGURE 5:**
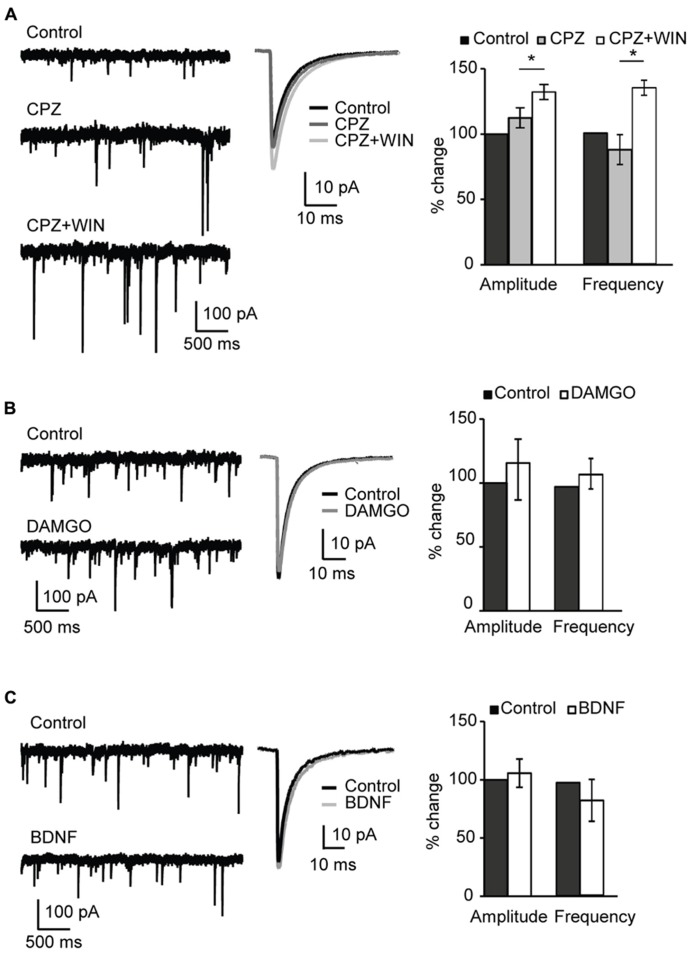
** Specificity of CB-dependent effects on sIPSCs. (A)** Blockade of TRPV1 receptors did not occlude the effect of WIN 55,212-2 on inhibitory neurotransmission. *Left*: Example traces of sIPSCs in control; in the presence of the TRPV1 receptor antagonist CPZ (caspazepine, 5 μM), and after perfusion of CPZ + WIN (caspazepine, 5 μM and WIN 55,212-2, 1 μM). *Middle*: Average sIPSC for the same conditions shown on the left. Black: control; dark gray: CPZ; light gray: CPZ + WIN. *Right*: Bar-plot summarizing the effect of CPZ and WIN on sIPSC amplitude and frequency. Black: control; gray: CPZ; white: CPZ + WIN. **(B)** Activation of opioid receptors did not affect sIPSC amplitude nor frequency. *Left*: Example traces of sIPSCs in control (top) and in the presence of the opioid receptor agonist DAMGO (0.1 μM, bottom). *Middle*: Average sIPSC for the same conditions shown on the left. Black: control; gray: DMAGO. *Right*: Bar plot summarizing the effect of DAMGO on sIPSC amplitude and frequency. Black: control; white: DAMGO. **(C)** Bath application of BDNF did not affect sIPSC amplitude and frequency. *Left*: Example traces of sIPSCs in control (top) and in the presence of BDNF (0.2 μM, bottom). *Middle*: Average sIPSC for the same conditions shown on the left. Black: control; gray: BDNF. *Right*: Bar plot summarizing the effect of BDNF on sIPSC amplitude and frequency. Black: control; white: BDNF. All data are expressed relative to their respective Control to show fold changes. Data are presented as mean ± SEM, statistical significance is indicated by * for *P* < 0.05.

Numerous studies confirmed that endocannabinoids, including anandamide, activate TRPV1 receptors (Ross, 2003; Pertwee, 2006; Gibson et al., 2008; Chavez et al., 2010). We therefore tested whether blockade of TRPV1 occluded the changes in mIPSC amplitude and frequency we observed with WIN and anandamide. TRPV1 is a non-selective ligand-gated cation channel with high Ca^2^^+^ permeability. Pharmacological blockade of TRPV1 receptors with capsazepine (5 μM, CPZ) did not affect amplitude and frequency of sIPSCs (amplitude, 112.4 ± 7.7%; p-*t*: *P* = 0.2; frequency 108.7 ± 11.4; p-*t*: *P* = 0.8; *n* = 8; **Figure 5A**, CPZ). Subsequent application of WIN 55,212-2 and capsazepine significantly increased sIPSC amplitude and frequency (amplitude, 132.2 ± 5.8%; p-*t*: *P* < 0.002; frequency 134.6 ± 5.7; p-*t*: *P* < 0.05; *n* = 8; **Figure 5A** CPZ + WIN). These data indicate that WIN 55,212-2-induced potentiation of inhibitory transmission did not result from unspecific activation of TRPV1 receptors.

We next assessed the involvement of opioid receptors in the potentiation of inhibitory drive. Numerous studies demonstrated an interaction between CB and opioid systems (Robledo et al., 2008; Spano et al., 2010). Besides producing common physiological effects such as inhibition of locomotor activity, sedation, analgesia or hypothermia CB and opioid receptors are colocalized in many tissues (Rodriguez et al., 2001; Salio et al., 2001; Pickel et al., 2004). Furthermore, direct receptor–receptor interaction of CB and opioid receptors was reported (Rios et al., 2006). To determine possible involvement of the activation of opioid receptors in the WIN-induced potentiation of inhibitory transmission, we examined the effect of the μ-opioid receptor agonist DAMGO (0.1 μM) on sIPSC amplitude and frequency. No significant changes were observed in either mIPSC amplitude or frequency (amplitude, 115.6 ± 23.8%; p-*t*: *P* = 0.9; frequency, 111.8 ± 12.2; p-*t*: *P* = 0.4; *n* = 6; **Figure 5B**). These data indicate that WIN 55,212-2 activation of μ-opioid receptors was not the mechanism leading to the increased inhibitory drive we observed in layer 4 of V1.

Lastly, we assessed the possible involvement of BDNF on the potentiation of inhibition we report. BDNF is a potent regulator of inhibitory synaptic transmission. Chronic exposure of hippocampal cultures to BDNF potentiated GABAergic inhibition (Bolton et al., 2000). In V1 over-expression of BDNF resulted in enhancement of GABAergic inhibition during developmentally restricted time periods and accelerated the maturation of the inhibitory network (Hanover et al., 1999; Huang et al., 1999). The distribution of BDNF TtrkB receptors in V1 replicates the distribution of CB1 receptors with a maximum expression in layers 2/3 and 5. The interaction between endocannabinoids and BDNF has been reported (Lemtiri-Chlieh and Levine, 2010). To verify possible involvement of BDNF receptors in the CB-induced potentiation of inhibitory transmission, we asked whether BDNF could mimic the effect of WIN 55,212-2 on sIPSCs. Bath application of BDNF (20 ng/ml; 0.8 nM) produced no significant change in either sIPSC amplitude or frequency (amplitude, 105.6 ± 12.2%; p-*t*: *P* = 0.2; frequency, 84.0 ± 18.5%; p-*t*: *P* = 0.3; *n* = 7; **Figure 5C**). Taken together these data demonstrate that, differently from what was observed in the rat hippocampus, the CB-dependent potentiation of inhibitory currents in L4 of V1 depends selectively on the activation of CB targets.

### ACTIVITY-DEPENDENT CHANGES IN INHIBITION

**FIGURE 6:**
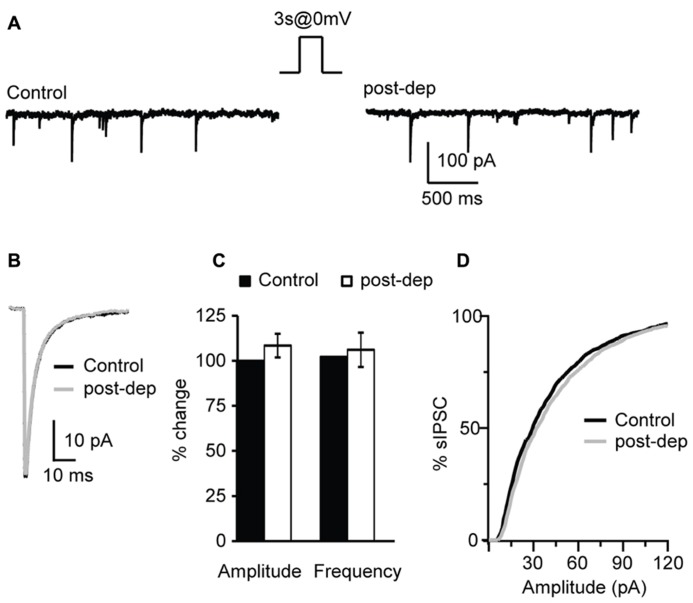
** Depolarization of L4 pyramidal neurons does not affect sIPSCs. (A)** Example traces of sIPSCs before (control) and immediately after the application of a 3 s long depolarizing pulse from -70 (holding potential) to 0 mV (post-dep). **(B)** Average sIPSC traces for the same experiment shown in **(A)**. Black: control; gray: post-dep. **(C)** Group data of sIPSC amplitude, and frequency before (black, control) and immediately after (white, post-dep) depolarization. Data are expressed relative to Control to show fold changes. **(D)** Cumulative distribution of sIPSC amplitudes in Control (black) and after depolarization (white, post-dep). Data are presented as mean ± SEM.

We have shown that bath application of CB receptors agonists can potentiate inhibitory synaptic transmission however, endocannabinoids are produced on demand and released in an activity-dependent-manner. Activity-dependent release of endocannabinoids can be induced by various patterns of activity, including steady depolarization of the post-synaptic neuron (Pitler and Alger, 1992; Ohno-Shosaku et al., 2001; Wilson and Nicoll, 2001), short burst (Brown et al., 2003; Fortin et al., 2004) or repetitive burst firing (Brenowitz et al., 2006). In this set of experiments we investigated the activity dependence of the CB-dependent potentiation of inhibition in L4 of V1. Post-synaptic depolarization has been shown to trigger the release of endocannabinoids via calcium-dependent mechanisms (Pitler and Alger, 1992; Ohno-Shosaku et al., 2001). We tested the possibility that activating L4 pyramidal neurons with a voltage step shifting the holding potential from -70 to 0 mV could affect the amplitude and frequency of sIPSCs recorded in the presence of AMPA and NMDA receptor blockers. In different sets of experiments depolarizing steps of different duration were tested: 1, 3, and 5 s. Recordings were performed before and immediately after depolarization for at least 3 min. Average sIPSC amplitude and frequency for events recorded in the 30 s prior and the 30 s after depolarization were compared. We did not detect significant changes in sIPSC amplitude (108.4 ± 6.6%, *n*= 10; p-*t*:* P *= 0.3) and frequency (104.5 ± 9.3%; p-*t*: *P *= 0.7; **Figures 6A–C**) after depolarization of any duration tested, indicating that depolarization in the absence of spiking activity does not mimic the potentiation of inhibition induced by WIN and AEA. The distributions of sIPSC amplitudes were not affected as well (**Figure 6D**). As no differences were observed using pulses of different duration, data were pooled.

Endocannabinoids were shown to modulate inhibitory neurotransmission in an activity-dependent fashion in cerebellum (Kreitzer and Regehr, 2001), hippocampus (Wilson and Nicoll, 2001; Chevaleyre and Castillo, 2003) and somatosensory cortex (Fortin et al., 2004). We tested whether AP firing of L4 pyramidal neurons could affect sIPSC amplitude and/or frequency. All these experiments were performed in the presence of AMPA and NMDA receptor blockers to prevent contributions of recurrent circuit activation to changes in inhibitory currents. A number of different AP paradigms were tested: firing of single AP, of a single burst of APs, and of repetitive bursts of AP. To induce single AP recorded pyramidal neurons were depolarized above threshold with a single 3 ms long suprathreshold depolarizing current step in current clamp. Amplitude and frequency of sIPSCs recorded from the activated neuron were quantified by comparing the last 150 sIPSC before and the first 150 sIPSC after induction. To examine short-term changes of inhibitory neurotransmission additional analysis was performed on the last 50 events before and first 50 events after induction. Firing of a single AP did not change mean sIPSC amplitude nor frequency (amplitude 104.0 ± 8.3%; p-*t* on average data: *P *= 0.6; K–S test on distributions: *P* = 0.9; frequency 102.4 ± 9.4%; p-*t* on average data: *P *= 0.7; K–S test on distributions: *P* = 0.9; *n*= 9). No short-term changes in inhibitory transmission were observed either (amplitude 103.0 ± 7.2%; p-*t*: *P *= 0.5; frequency 105.8 ± 8.1%; p-*t*: *P *= 0.7; *n*= 9). CB release depends on activation of intracellular signaling mechanisms, thus it is possible that the lack of changes in sIPSCs may be due to failure of a single AP to effectively trigger CB release. We asked whether a burst of APs could potentiate inhibition onto the activated neuron. To do that, recorded neurons were made to fire 20 APs by injecting 20 – 3 ms long suprathreshold depolarizing steps at 50 Hz. Our data show that a single burst of 20 APs at 50 Hz was not sufficient to alter sIPSC amplitude (96.1 ± 3.3%; p-*t* on average data:* P *= 0.3; K–S test on distributions: *P* = 0.6; *n*= 7) and frequency (107.8 ± 6.7%; p-*t *on average data:* P *= 0.2; K–S test on distributions: *P* = 0.9). No short-term changes of sIPSC were observed as well (amplitude: 88.8 ± 11.4%; p-*t*: *P *= 0.2; frequency: 97.7 ± 11.8%; p-*t*: *P *= 0.4; *n*= 7).

**FIGURE 7:**
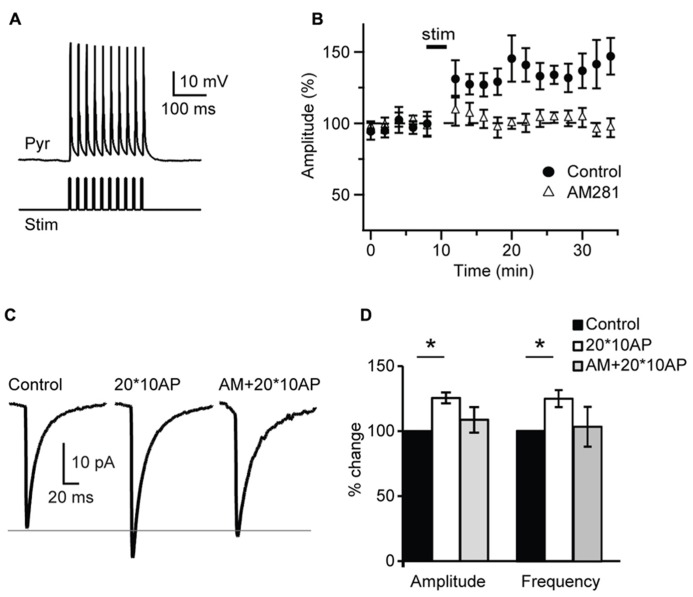
** Repetitive burst firing of layer 4 pyramidal neurons induces endocannabinoid-mediated potentiation of inhibition. (A)** Response of pyramidal neuron (Pyr) to the injection of 10 – 3 ms long depolarizing current pulses (Stim). **(B)** Repetitive burst firing of layer 4 pyramidal neuron (20 bursts of 10 action potentials at 50 Hz repeated at 0.1 Hz) potentiates sIPSC amplitude. The potentiation is prevented by AM281 (1 μM). Stim: application of induction paradigm. **(C)** Average sIPSCs before (control) and after burst firing in standard ACSF (20*10AP) and in the presence of AM281 (AM + 20*10AP). Traces are from the cell shown in **(A)**. **(D)** Group data of sIPSC amplitude and frequency before and after burst firing. Black: control; white: 20*10AP; gray: AM + 20*10AP. All data are expressed relative to Control to show fold changes. Data are presented as mean ± SEM, statistical significance is indicated by * for *P* < 0.05.

The lack of activity-dependent changes in inhibitory drive observed after a burst of APs may be due insufficient release of endocannabinoids or to failure to drive activity-dependent synthesis of CB. Thus we tested whether repetitive bursting may be more effective. 20 bursts of pyramidal neuron APs organized in 10 – 3 ms long suprathreshold depolarizing pulses at 50 Hz each delivered at 0.1 Hz, a protocol known to induce plasticity quite effectively at L4 recurrent excitatory synapses (Wang et al., 2012), was applied in the attempt to potentiate sIPSC amplitude and frequency in the presence of glutamate receptors blockers. Repetitive pyramidal neurons bursting did not affect passive membrane properties like series resistance (p-*t*:* P* = 0.4,), input resistance (p-*t*:* P* = 0.6) and resting membrane potential (p-*t*:* P* = 0.9). In contrast repetitive burst firing potentiated inhibitory transmission onto the recorded pyramidal neuron, increasing both sIPSC amplitude and frequency (amplitude: 125.5 ± 4.2%; p-*t *on average data:* P *< 0.05; K–S test on distributions: *P *< 0.01; frequency: 124.7 ± 6.5%; p-*t* on average data:* P *< 0.05; K–S test on distributions: *P *< 0.01; *n*= 7; **Figure 7**). Bath application of the CB receptor antagonist AM 281 (1 μM) prevented potentiation of sIPSC amplitude (*n*= 7, K–S test on distributions: *P* = 0.9; p-*t* on average data:* P* = 0.08) and frequency (K–S test on distributions:* P* = 0.9; p-*t* on average data:* P* = 0.1) induced by repetitive bursting. Thus, repetitive L4 pyramidal neuron bursting can induce a CB-dependent form of postsynaptic-dependent long term potentiation of inhibition (POSD-LTPi).

As most reported CB-dependent forms of plasticity require calcium signaling we asked whether POSD-LTPi may also be calcium-dependent. In a set of experiments the calcium chelator BAPTA (5 mM) was added to the internal solution used for patch clamp recordings. Once a stable recording configuration was obtained, a 10 min baseline was acquired and then the repetitive burst firing paradigm was applied. Amplitude and frequency of sIPSCs recorded before and after induction were compared. Intracellular BAPTA impaired POSD-LTPi induction (amplitude: 105.8 ± 8.8% of baseline; K–S test on distributions:* P* = 0.7; p-*t* on average data: *P* = 0.51; frequency: 97.8 ± 6.0% of baseline; K–S test on distributions:* P* = 0.9; p-*t* on average data: *P* = 0.74; *n* = 8), indicating that this form of inhibitory plasticity is calcium-dependent.

These data indicate that that CB signaling can increase inhibitory drive onto active L4 pyramidal neurons, possibly limiting their excitability. This form of plasticity is induced by repetitive pyramidal neuron bursting in the absence of glutamatergic receptors activation, but is not induced by steady-state depolarization, and is calcium-dependent. By increasing inhibitory drive onto a bursting pyramidal neuron, this form of CB-dependent inhibitory potentiation may be a novel mechanism for the homeostatic regulation of V1excitability.

### CANNABINOID-DEPENDENT POTENTIATION OF INHIBITION IS DEVELOPMENTALLY REGULATED

**FIGURE 8:**
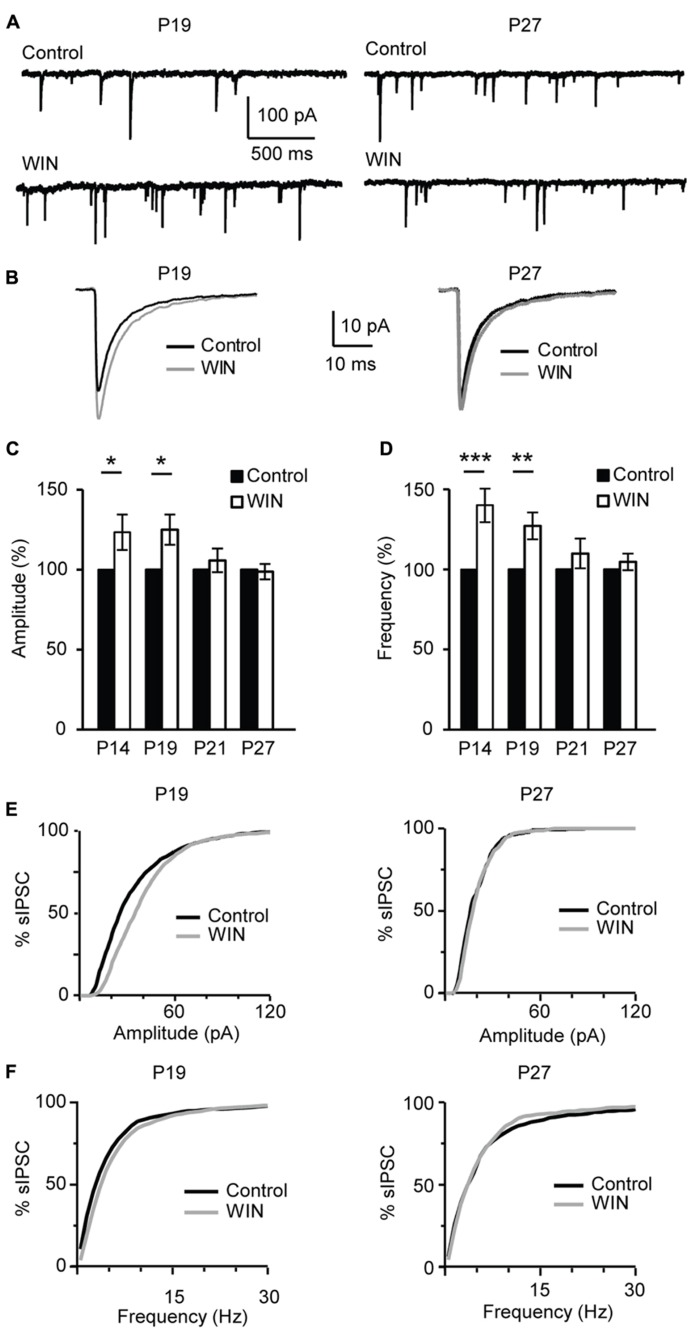
** CB-dependent inhibitory potentiation is developmentally regulated. (A)** Example traces showing sIPSCs recordings for Control and following WIN application (WIN 55,212-2, 1 μM) at P19 and P27. **(B)** Average sIPSCs for the conditions shown in **(A)**. Black: control; gray: WIN 55,212-2. **(C**,**D)** Bar plot summarizing the effect of WIN 55,212-2 on sIPSC amplitude **(C)** and frequency **(D)** by age groups P14 (immediately after eye opening), P19 (pre-critical period), P21 (onset of the critical period), P27 (peak of the critical period). Black: control; white: WIN 55,212-2. Amplitude and frequency are represented relative to the Control for each age group to show fold changes. **(E)** Cumulative distribution of sIPSC amplitudes for control (black) and WIN (gray) at P19 (left) and P27 (right). **(F)** Cumulative distribution of sIPSC frequencies for control (black) and WIN (gray) at P19 (left) and P27 (right). Data are presented as mean ± SEM, statistical significance is indicated by * for *P* < 0.05, ** for *P* < 0.001, *** for *P *< 0.001.

During postnatal development gradual increases in CB1 receptor distribution were detected in primary somatosensory cortex (S1) and V1 (Deshmukh et al., 2007; Yoneda et al., 2013). In addition, the expression of CB1 receptors in V1 and S1 was developmentally restricted (Yoneda et al., 2013) supporting the view that endocannabinoid signaling may be limited to specific developmental windows. To determine whether the CB-dependent potentiation of inhibition we report is expressed throughout life or is specific for the first few weeks of postnatal development, we examined its inducibility in the window from eye opening to the peak of the critical period for visual cortical plasticity. This time window was chosen based on recent findings showing developmental regulation of CB-dependent depression in the superficial layers of V1 (Liu et al., 2008; Jiang et al., 2010b). We compared the effect of bath application of CB agonists on sIPSC amplitude and frequency onto L4 pyramidal neurons in slices obtained from mice from four age groups P14 (immediately after eye opening), P19 (pre-critical period, see data in **Figures 1–7**), P21 (onset of the critical period), P27 (peak of the critical period; **Figure 8**). In slices prepared from P14 mice bath application of WIN 55,212-2 (1 μM) significantly increased sIPSC amplitude to 121.6 ± 11.1% of baseline and frequency to 140.0 ± 10.5% of baseline (*n* = 13; amplitude: K–S test on distributions: *P* < 0.01; p-*t* on average data: *P* < 0.05; Frequency: K–S test on distributions:* P* < 0.001; p-*t* on average data: *P* < 0.001; **Figures 8C,D**). These changes were similar to those observed at P19 Figures 1 and 8A–E). Differently, at P21 and P27 bath application of WIN 55,212-2 did not affect average sIPSC amplitude nor frequency (P21, amplitude, 105.8 ± 7.4% of baseline; K–S test on distributions: *P* = 0.9; p-*t* on average data: *P* = 0.1; frequency 109.0 ± 9.3% of baseline; K–S test on distributions: *P* = 0.9; p-*t* on average data: *P* = 0.1; P27, amplitude: 98.7 ± 4.8% of baseline; K–S test on distributions: *P* = 0.28; p-*t* on average data: *P* = 0.6; frequency, 104.7 ± 5.2% of baseline; *n* = 18; K–S test on distributions: *P* = 0.81; p-*t* on average data: *P *= 0.7; **Figures 8A–E**).

**FIGURE 9:**
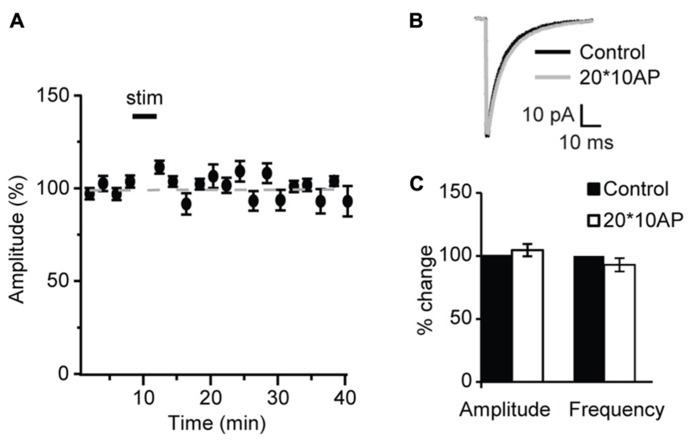
** After the onset of the critical period repetitive burst firing of layer 4 pyramidal neurons does not affect sIPSCs. (A)** Burst firing of pyramidal neuron (20 bursts of 10 action potentials at 50 Hz repeated at 0.1 Hz) did not affect sIPSC amplitude at P27. Stim: application of induction paradigm. **(B)** sIPSC traces averaged across 150 events before (black) and after (gray) burst firing. **(C)** Group data of sIPSC average amplitude and frequency before (black bar) and after (white bar) repetitive pyramidal neuron bursting. All data are expressed relative to Control to show fold changes. *p < 0.05.

Similarly, POSD-LTPi was induced only in the pre-critical period. As shown in **Figure 9**, repetitive pyramidal neuron bursting in the presence of AMPA and NMDA receptor blockers did not change sIPSC amplitude nor frequency in acute slices from P27 mice (**Figure 9**; amplitude: 97.1 ± 8.5 of control; K–S test on distributions:* P* = 0.9; p-*t* on average data: *P* = 0.84; frequency: 92.8 ± 4.8 of control; K–S test on distributions:* P* = 0.4; p-*t* on average data: *P* = 0.6; *n* = 5) strongly suggesting that WIN-dependent sIPSC potentiation and POSD-LTPi share similar induction mechanisms. This data also indicate that CB-dependent POSD-LTPi is restricted to a short period during postnatal development. The transition from the pre-critical to the critical period for visual cortical plasticity is marked by a loss of CB-dependent POSD-LTPi onto L4 pyramidal neurons.

### CB-DEPENDENT POTENTIATION OF INHIBITION IS EXPERIENCE-DEPENDENT

**FIGURE 10:**
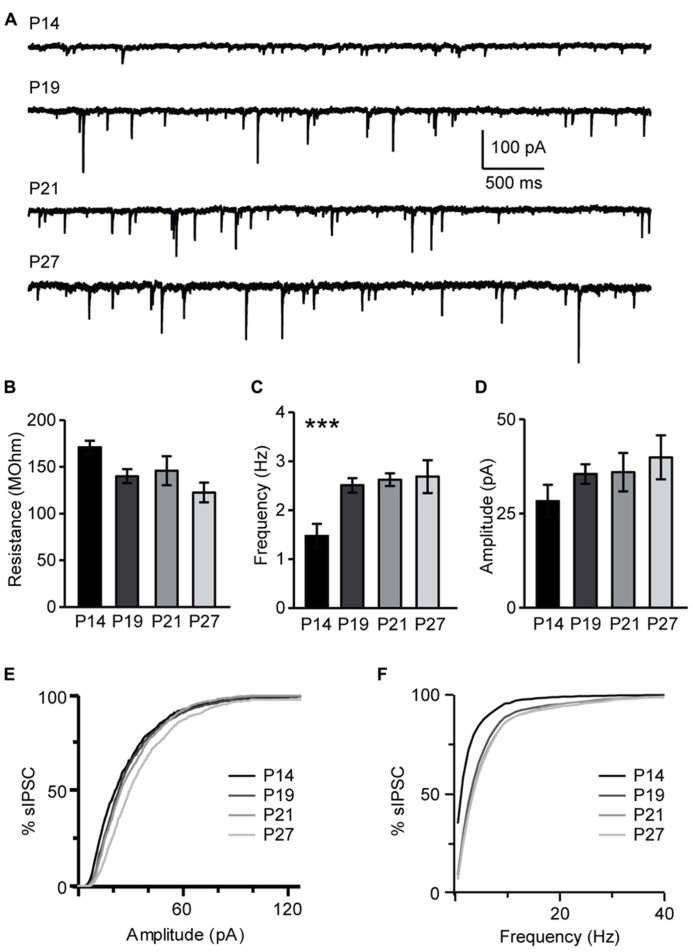
** Developmental changes in sIPSC amplitude and frequency. (A)** Representative examples of sIPSCs recordings for different age groups. **(B)** Group data for average resting input resistance in the different age groups. **(C)** Average sIPSC frequency for all age groups. **(D)** Average sIPSC amplitude for all age groups. For **(B–D)**: black bar: P14; dark gray bar: P19; light gray bar: P21; lightest gray bar: P27. **(E)** Cumulative distribution of sIPSC amplitudes for the different age groups. **(F)** Cumulative distribution of sIPSC frequencies for the different age groups. For **(E,F)**: black: P14; dark gray: P19; light gray: P21; lightest gray: P27. Data are presented as mean ± SEM, statistical significance is indicated by *** for *P* < 0.001.

The maturation of the GABAergic system is critical for the onset of the critical period for visual cortical plasticity (Fagiolini and Hensch, 2000). Developmental changes of inhibitory synaptic transmission parallel the maturation of the endocannabinoids system. The restriction of pyramidal neurons capacity for CB-dependent inhibitory plasticity to the time immediately following eye opening suggests that this form of plasticity may be sensitive to visual experience. We investigated this directly by determining first how sIPSC properties may change during the developmental window under study and then by assessing the role of the time of eye opening on the capacity for CB-dependent inhibitory plasticity. In L4, sIPSC frequency increased significantly from P14 to P19 and then remained stable (P14, 1.47 ± 0.25 Hz, *n* = 13; P19, 2.51 ± 0.20 Hz, *n* = 13, *P* < 0.001; P21, 2.62 ± 0.13 Hz, *n* = 13, *P* < 0.001; P27, 2.93 ± 0.33 Hz, *n* = 13, one-way ANOVA with *post hoc* Tukey–Kramer test for multiple comparisons:* P* < 0.001; K–S test on distributions: P14–P19: *P* < 0.001; P19–P21: *P* < 0.002; P21–P27: *P* = 0.33; **Figures 10A,C,F**). Differently, the average sIPSC amplitude did not change significantly from P14 to P27 (P14, 28.3 ± 4.3 pA, *n* = 13; P19, 35.5 ± 2.6 pA, *n*= 13; P21, 36.0 ± 5.1 pA, *n* = 13; P27, 35.0 ± 2.4 pA, *n* = 13; one-way ANOVA with *post hoc* Tukey–Kramer test for multiple comparisons:* P* = 0.4); while the distribution of sIPSC amplitudes showed significant differences after P19, at the transition from the pre-critical to the critical period (K–S test on distributions: P14–P19: P = 0.08; P19–P21: *P* < 0.001; P21–P27: *P* < 0.001; **Figures 10B,D,F**). Thus, GABAergic sIPSCs in L4 of V1 progressively increase in frequency during development and reach a steady-state at P19, when CB-dependent potentiation of inhibition is still effectively induced and before the onset of the critical period for visual cortical plasticity. The amplitude of sIPSCs increased after the onset of the critical period, consistent with previous findings that inhibitory synapses maturation in mice V1 extends beyond the first three postnatal weeks (Morales et al., 2002). Together this data suggest that the capacity for CB-dependent inhibitory plasticity in L4 is not occluded by the maturation of GABAergic synaptic transmission. Instead, the process of maturation and capacity for CB-dependent potentiation of inhibition are parallel processes regulating inhibitory synaptic drive onto L4 pyramidal neurons during postnatal development.

As CB-dependent potentiation of inhibition is limited to the third week in development we tested the hypothesis that it may be involved in regulating the excitability of L4 pyramidal neurons at eye opening, when V1 neurons transition from being activated by spontaneous activity, to being driven by visual stimuli. To do that, we delayed eye opening for 13 days with binocular eyelid sutures started at P13, when the eyes of the mouse are still closed, and maintained to P27. Recordings were performed at P27 ± 1 day, close to the peak of the critical period, and according to our findings, a time in which CB signaling does not affect sIPSC amplitude and frequency in L4 of V1. Analysis of baseline sIPSC amplitude and frequency showed no significant differences between littermates that were binocularly deprived and those whose eyes were allowed to open (amplitude: 106.4 ± 7.3%; K–S test on distributions: *P* = 0.15; unpaired *t*-test on average data:* P* = 0.6; frequency: 111.4 ± 12.5%; K–S test on distributions: *P* = 0.42; unpaired *t*-test on average data: and *P* = 0.5; *n* = 13). These data support the interpretation that the developmental changes in amplitude and frequency of sIPSCs in L4 of V1 occur independently of visual drive.

**FIGURE 11:**
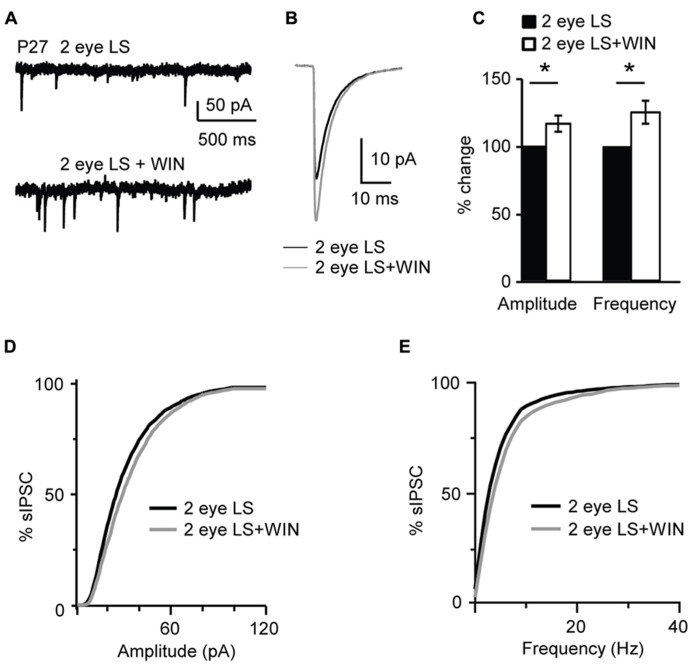
** Binocular lid suture delays the window for CB-dependent potentiation of inhibition. (A)** Example traces of sIPSCs recordings in Control and after application of WIN (WIN 55,212-2, 1 μM) in animal whose time of eye opening was delayed from P13 to P27 with binocular eyelid suture (2 eye LS). Recording were performed at P27 – a time in development in which sIPSCs from normally reared mice show no sensitivity to WIN. **(B)** Average sIPSCs traces for the conditions shown in **(A)**. Black: 2 eye LS; gray: 2 eye LS + WIN. **(C)** Bar plot summarizing the effect of WIN 55,212-2 on average sIPSC amplitude and frequency in binocularly deprived mice. Black: 2 eye LS; white: 2 eye LS + WIN. Data are expressed relative to Control to show fold changes. **(D)** Cumulative distribution of sIPSC amplitudes for control (2 eye LS) and WIN application (2 eye LS + WIN) in binocularly deprived mice. **(E)** Cumulative distribution of sIPSC frequencies for control (2 eye LS, black) and WIN application (2 eye LS + WIN, gray) in binocularly deprived mice. Data are presented as mean ± SEM, statistical significance is indicated by * for *P* < 0.05.

In contrast, bath application of WIN 55,212-2 (1 μM) significantly increased amplitude and frequency of sIPSC recorded from slices obtained from P27 binocularly deprived mice (amplitude: 116.1 ± 5.9%; K–S test on distributions: P < 0.001; p-*t* on average data: P < 0.01; frequency: 125.0 ± 8.4% of baseline; K–S test on distributions: P < 0.001; p-*t* on average data: P < 0.007; *n*= 15; **Figure 11**), while it did not affect sIPSC amplitude of the non-deprived littermates. These data demonstrate that the capacity of L4 pyramidal neurons to induce CB-dependent potentiation of inhibition is independent of the maturation of GABAergic synapses, but is regulated by the time of eye opening.

## DISCUSSION

We report a novel form of inhibitory synaptic plasticity at GABAergic synapses onto pyramidal neurons in the input layer of V1. This form of potentiation of inhibition is induced by repetitive burst firing of L4 pyramidal neurons, and affected inhibitory drive onto the bursting neuron globally by increasing amplitude and frequency of sIPSCs. The potentiation of inhibition we report depends on CB-signaling; requires intracellular calcium signals, and pyramidal neuron spiking. Furthermore, it is effectively induced only in the developmental window from eye opening to the onset of the critical period. The capacity of inhibitory inputs to undergo this form of inhibitory potentiation is tightly regulated by the time of eye opening. We propose that this form of CB-dependent potentiation of inhibitory drive may play a homeostatic function at a time in development in which V1 neurons transition from being activated by spontaneous retinal activity to being driven by patterned vision.

### ENDOCANNABINOID RECEPTORS AND SYNAPTIC PLASTICITY

Regulation of CB signaling is crucial for the healthy postnatal development of neural circuits. It controls the development of dendritic structures in specific neuron types, as well as the fasciculation and pathfinding of both corticothalamic and thalamocortical axons (Wu et al., 2010). Altered CB signaling during an early critical period disrupts whisker map development and alters experience-dependent plasticity in L4 of S1 (Li et al., 2009). Furthermore, modulation of CB signaling contributes to memory formation, development of addictive behaviors, regulation of pain, central regulation of metabolism and food intake, and has been associated with a number of neurological disorders (Chaperon and Thiebot, 1999).

At the cellular and circuit level, CB signaling has predominantly been associated with induction of long term depression both at excitatory and inhibitory synapses (Chevaleyre and Castillo, 2004; Hashimotodani et al., 2007; Castillo et al., 2012). These data suggest that CB-dependent induction of long term changes in synaptic strength may be one of the circuit mechanisms mediating the persistent effects of CB signaling on sensory perception and behavior. Recent work demonstrated that, besides long term synaptic plasticity, CB signaling mediates self down-regulation of cortical excitatory and inhibitory neurons, suggesting an important role for CB signaling in the maintenance of network stability (Bacci et al., 2003, 2004). All these data demonstrated the role of CB signaling in decreasing synaptic drive. However, recent work showed that both in the goldfish nervous system and in the mammalian hippocampus CB-signaling may also mediate up-regulation of inhibition (Cachope et al., 2007; Hofmann et al., 2011).

Our findings indicate that in L4 of V1 CB signaling contributes to the regulation of pyramidal neurons excitability through a form of activity-dependent potentiation of inhibitory drive that is independent of recurrent circuit activation. In this study we explored the parameter space for induction, the developmental regulation, and begun investigating the signaling mechanisms involved in this form of inhibitory plasticity.

The form of inhibitory potentiation reported here presents significant differences from previous reports. While in the hippocampus the only evidence for CB-dependent potentiation of inhibition is independent of AP firing, our data show that in L4 of V1 bursting of the post-synaptic pyramidal neurons is necessary for successful induction, while depolarization in the absence of spiking is ineffective. The effect of CB-signaling was specific to inhibitory inputs as no changes in mEPSCs onto pyramidal were induced by CB agonists.

The mechanisms for this novel form of inhibitory potentiation show some similarity to those involved in the well studied forms of CB-dependent synaptic depression (Chevaleyre and Castillo, 2003) as it is calcium-dependent and appears to have a presynaptic site of expression as supported by experiments showing significant increases in sIPSC frequency following bath application of CB agonists or POSD-LTPi induction, and CB-dependent increases in reliability of evoked IPSCs in response to repetitive stimulation. We also observed a significant increase in sIPSC amplitude. A possible mechanism justifying both changes in frequency and amplitude of sIPSCs could be, as found in the hippocampus, that GABA_A_ receptors are not saturated by activity-independent release (Cohen et al., 2000). Thus, increasing GABA release could in principle lead to increased frequency and amplitude of sIPSCs by increasing GABA binding. While the saturation of GABA_A_ receptors by single release events has not been investigated in V1, a similar mechanism could be at play at the inhibitory synapses we studied. Additional, yet unidentified, post-synaptic signaling mechanisms activated by CB agonists could also be involved. Further studies will be needed to fully identify the site/sites of expression and biochemical pathways involved in this process.

### CB SIGNALING AND VISUAL CORTICAL DEVELOPMENT

L4 neurons loose capacity for CB-dependent potentiation of inhibition and POSD-LTPi after the onset of the classical critical period for visual cortical plasticity. The capacity for CB-dependent inhibitory potentiation is not occluded by GABAergic synapses maturation, but is regulated by the time of eye opening. Our data show that 13 days binocular deprivation by eyelid suture do not affect the developmental changes in sIPSC amplitude and frequency, but selectively affect the capacity for CB-dependent modulation of inhibition and POSD-LTPi induction. The developmental time course of this form of plasticity is consistent with the age-dependent regulation of CB1 receptors expression in L4. After the onset of the critical period CB signaling does not affect inhibitory synapses in L4, however, it can decrease the strength of inhibitory synapses in layer 2/3 (Jiang et al., 2010b), emphasizing the layer specificity of the events involved in the maturation and refinement of V1.

In rodents eye opening is determined by the loss of a semi-transparent barrier reducing the direct activation of retina by natural light stimuli at the early stages of postnatal development. At eye opening patterned vision elevates retinal activity transiently (Stasheff et al., 2011), possibly increasing the activation of visual cortical circuits. Consistent with this, both evoked and spontaneous AMPA and NMDA currents were shown to increase 12 h after eye opening in visual neurons of superior colliculus (Lu and Constantine-Paton, 2004). The increased excitatory drive onto visual neurons occurs in parallel with, and probably depends on, the increase of PSD-95 levels of expression. Although the immediate effects of eye opening on V1 neurons have not been directly investigated, changes in PSD-95 protein levels similar to those recorded in the superior colliculus were observed as early as 6 h after eye opening (Yoshii et al., 2003). The increase in retinal activity after eye opening could overexcite visual cortical circuits. As in normal mice the transition to patterned vision does not result in instability of the visual cortical circuit, homeostatic mechanisms may be in place to regulate of circuit excitability and avoid the occurrence of pathological states. CB-dependent regulation of inhibition was proposed as a homeostatic mechanism for the regulation of hippocampal excitability during postnatal development (Bernard et al., 2005). Repetitive bursting of pyramidal neurons induced CB-dependent POSD-LTPi, suggesting that in V1 CB-signaling plays a homeostatic role by potentiating global inhibitory drive onto highly active L4 pyramidal neurons. Besides regulating L4 pyramidal neurons excitability, the tight developmental regulation of POSD-LTPi suggests that it may also contribute to the development of visual neurons functions that are known to become established prior to the onset of the critical period. The effect of CB on the circuit is specific to inhibitory inputs onto pyramidal neurons and is restricted to the pre-critical period for visual cortical plasticity. Our data show that there was no CB-dependent inhibitory plasticity in L4 in the critical period, consistent with previous findings (Liu et al., 2008). When looked in the context of previously published work our data indicate that during the developmental window from eye opening to the onset of the critical period the circuit in L4 is endowed with a complex set of mechanisms that may regulate circuit function: CB-dependent POSD-LTPi at eye opening (current work), as well as a combination of scaling up of excitation and scaling down of inhibition in response to brief monocular deprivation (Maffei et al., 2004). These forms of plasticity are not observed after the onset of the critical period, suggesting that the circuit in L4 undergoes a complex series of maturation events that affect not only its capacity for plasticity, but also the mechanisms involved in the maintenance of stable states of excitability. Delaying the time of eye opening reopens the window of sensitivity for CB-dependent inhibitory self-potentiation, suggesting that this form of plasticity is tightly regulated by the transition from spontaneous to visually evoked activity.

Our results, together with previous work, also suggest that CB signaling may affect L4 synapses differently depending on the source of the activity: in our experiments repetitive bursting of L4 pyramidal neurons was necessary to induce the CB-dependent POSD-LTPi. In contrast, activation of white matter afferents onto L4 neurons did not affect the amplitude and short-term dynamics of feedforward evoked IPSCs even in the presence of CB receptor agonists (Jiang et al., 2010a). Our data suggest at the time of eye opening intracortical spontaneous activity may still contribute extensively to circuit refinement and part of the contribution of intracortical activity may depend on the presence of a strong sensitivity of L4 neurons to changes in the state of excitability of different elements of the local microcircuit. This could explain the apparent inconsistencies between the effect of CB agonists on IPSCs evoked by white matter stimulation and our study. CB signaling likely plays a complex role in brain circuit wiring not only by modulating axon pathfinding and prenatal wiring of neural circuits, but also by regulating synaptic strength of excitatory and inhibitory synapses postnatally with a high degree of specificity for different components of specific circuits, sign of plasticity, and developmental window.

## Conflict of Interest Statement

The authors declare that the research was conducted in the absence of any commercial or financial relationships that could be construed as a potential conflict of interest.
